# Workplace violence against security personnel at a university hospital in Egypt: a cross-sectional study

**DOI:** 10.12688/f1000research.23252.1

**Published:** 2020-05-11

**Authors:** Ahmed A. Albadry, Abdel-Hady El-Gilany, Hala Samir Abou-ElWafa

**Affiliations:** 1 Industrial Medicine and Occupational Health, Public Health & Community Medicine Department, Faculty of Medicine, Mansoura University, Mansoura, Egypt; 2 Public Health and Preventive Medicine, Public Health and Community Medicine Department, Faculty of Medicine, Mansoura University, Mansoura, Egypt

**Keywords:** Hospital violence, Security personnel, occupational exposure

## Abstract

**Background: **Violence is common among security personnel. To the best of the authors' knowledge no recent studies have investigated this problem. This study aimed to estimate the prevalence and associated factors of violence against hospital security personnel and describe circumstances of violence, type of perpetrators, and victims’ response.

**Methods: **In total, 170 security personnel from a university hospital in Egypt were recruited in this cross-sectional study. Data were collected using the Arabic version of a questionnaire developed by the International Labour Office.

**Results:** The majority (87.3%) of security personnel reported violence exposure in the past year. Being a woman and working more than 5 years were independent predictors of violence exposure. The commonest forms of physical violence were pushing and beating. Verbal abuse and threats were the commonest emotional violence. Patients and their relatives/friends were the commonest perpetrators of violence.

**Conclusions:** Violence is common among hospital security personnel in this setting. Adequate training and recruitment of more security personnel may contribute to decreasing violence.

## Introduction

Workplace violence (WPV) is well-defined as any act or danger of physical violence, harassment, bullying, or other disruptive activities at the workplace that may result in physical or emotional problems ^1^. The literature and news show that violence is common globally and occurs every day in various public spheres, including the health system ^2^.

Healthcare violence reflects violence in society in general. However, violence contradictory to social expectations that hospitals are sites that denote security, care, sympathy, and lifesaving. It is hard to acknowledge that violent incidents occur commonly in hospitals and in some situations physical violence in hospitals can be exceedingly dangerous ^3^.

Healthcare WPV is an underestimated and constant problem that has been widely overlooked. Many healthcare organizations and institutions that are considered safe resorts are now facing “steadily growing rates of crime, including violent criminalities such as stabbing, rape, and homicide” as stated by Phillips ^4^. According to the US Agency of Labor Statistics 2007 report, violence occurs more habitually in healthcare and social support organizations than in any other labor force segment and is responsible for 60% of all nonfatal assaults within this segment ^5^.

The majority of research studies have revealed that following an incident of WPV, there are high rates of lost working days, burnout, and dissatisfaction, in addition to decreased feelings of wellbeing among staff members ^6, 7^. In return, fright at work has even driven some healthcare personnel to protect themselves by carrying weapons, mostly firearms or knives ^8^.

Hospital security officers are required to protect the safety of healthcare workers, hospital guests, and patients. They are asked to assist in control of violent situations and are consequently at an elevated risk to tolerate violence-associated incidences and are therefore recognized as a group at high risk of being attacked ^9^. They are often disregarded in the literature concerning healthcare violence-related incidences. The comparatively few studies that do involve hospital security staff demonstrate that these workers are among groups with the highest rates of violence-related incidences within the healthcare setting ^10– 12^.

The significance of policies and security staff training has been accepted and various hospitals offer limitation tools and weapons to security personnel. Such establishment, chiefly of non-lethal intermediary weapons (e.g. conducted electrical weapons) or lethal weapons (e.g. handguns) has been conflicted owing to the moral, legal, and financial issues related to the well-being and safety of patients and staff in hospitals ^13, 14^.

WPV directed towards hospital security staff has seldom been studied in developing countries including Egypt; thus, the actual magnitude of the problem is unknown. The purposes of this study are to estimate the prevalence and associated issues of different types of violence against hospital security personnel, conditions of violence, category of perpetrators, and victims’ reaction in a university hospital setting.

## Methods

### Study description and participants

This descriptive cross-sectional study was conducted among formal security personnel at a university hospital from September 1, 2018 to October 31, 2018.

All formal security personnel were the target population. The inclusion criteria were permanent or temporary workers employed for 1 year or more.

Approval for the study was obtained from the Institutional Research Board at the university hospital (proposal number: R.18.07.236). The name of the university hospital has been blinded to protect anonymity of participants. Written informed consent of study participants to participate willingly in the study with the right to withdraw from the research at any time was obtained with a guarantee of confidentiality and anonymity of the information.

### Data collection

Workers fulfilling the eligibility criteria were interviewed at their workplace at the beginning of the workday before their work shift after arrangement with their direct supervisor in the supervisor room. The English version of the questionnaire developed by the International Labor Office, International Council of Nurses, World Health Organization, and Public Services International (2003) regarding WPV in the health sector was used in Arabic (English version available here: https://www.who.int/violence_injury_prevention/violence/interpersonal/en/WVquestionnaire.pdf. A modified Arabic version of this questionnaire was developed and tested for validity and reliability in a previous study in Saudi Arabia ^15^. The questionnaire was used to collect the following information: demographics and workplace characteristics; WPV in the past year; its nature, frequency, response, consequences, and satisfaction of incident handling; policies, and strategies to deal with the incident.

### Data analysis

Data were statistically analysed using the Statistical Package for Social Sciences (SPSS) version 20. Data were presented in the form of numbers and %. Chi-squared test was used to determine significant risk factors of violence. Multivariate logistic regression analysis using forward Wald method was run to identify significant independent predictors of violence. Crude and adjusted odds ratios and their 95% confidence intervals were considered. P≤0.05 was considered statistically significant.

## Results

The questionnaire was completed by 150 out of 170 legible security personnel (response rate 88.2%) who reported a total number of 553 workplace events in the past year.

The majority (87.3%) of security personnel reported exposure to violence in the past year. Being a woman and working for over 5 years were independent predictors of violence exposure (adjusted odds ratio (AOR) = 7.7 and 10.1, respectively; Table 1).

**Table 1.** Prevalence of workplace violence against security personnel at a university hospital in Egypt and its predictors during past year.

	Total, n	Violence experienced, n (%)	Crude odds ratio (95%)	Adjusted odds ratio (95%)
**Total**	150	131 (87.3)	(82.0-92.7)	
**Gender** Male Female	94 56	77 (81.9) 54 (96.4)	1(r) 6.0 (1.3-26.9) **	1(r) 7.7(1.6-336.3) **
**Age, years** <35 ≥35	93 57	75 (80.6) 56 (98.2)	1(r) 13.4 (1.7-103.7) **	
**Duration of work, years** 1–5 >5	67 83	51 (76.1) 80 (96.4)	1(r) 8.4 (2.3-30.2) ***	1(r) 10.1(2.7-37.4) ***
**Previous training** No Yes	68 82	57 (83.8) 74 (90.2)	1(r) 1.8 (0.7-4.7)	
Constant Model χ2 % correctly predicted				0.6 24.3 *** 87.3

r=reference group. **P≤0.01 ***P≤0.001.

Table 2 shows that the commonest suggestions from participants to prevent violence were training (73.3%) and assigning a large number of personnel (62.7%), while Table 3 shows that the commonest forms of physical violence reported by personnel were pushing (24.8%), beating (18.6%) and throwing objects (12.3%). The *threat* of physical force and verbal abuse were the commonest emotional violence experienced by participants (23.7% and 20.4%; respectively). Patients’ relatives/friends and patients themselves were the commonest perpetrators of violent events (72.5% and 23.3%; respectively).

**Table 2.** Prevention tactics for workplace violence reported by security personnel at a university hospital in Egypt (n=150).

Measure to prevent violence	Won't help	Will help to some extent	Will help
	n (%)		
Train workers on how to prevent violence.	15 (10.0)	25 (16.7)	110 (73.3)
Assign large numbers of security personnel over different work shifts.	15 (10.0)	41 (27.3)	94 (62.7)
Create a commission responsible for protection of employees in the course of duty.	22 (14.7)	41 (27.3)	87 (58.0)
Change work environment and flow.	16 (10.7)	48 (32.0)	86 (57.3)
Familiarize employees with their legal rights if they are subjected to violence in their workplace.	23 (15.3)	42 (28.0)	85 (56.7)
Define prohibitions within procedures for admission of patients.	26 (17.3)	39 (26.0)	85 (56.7)
Create a policy for care of violence victims.	23 (15.3)	43 (28.7)	84 (56.0)
Install violence alarm system.	36 (24.0)	33 (22.0)	81 (54.0)
Introduce a practical procedure for how to deal with the reality of violence that occurs in the workplace.	41 (27.3)	31 (20.7)	78 (52.0)
Install metal detectors at all entrances.	44 (29.3)	31 (20.7)	75 (50.0)
Improve the level of lighting in all sections of the health facility.	33 (22.0)	43 (28.7)	74 (49.3)
Use the closed-circuit television system.	51 (34.0)	32 (21.3)	67 (44.6)
Create a guide to each section of the health facility that determines the different forms of violence against workers and how to deal with this.	30 (20.0)	58 (38.7)	62 (41.3)
Other administrative measures.	36 (24.0)	56 (37.3)	58 (38.7)

**Table 3.** Types of violence and the main perpetrators from 553 workplace violence events reported by security personnel at a university hospital in Egypt.

	n (%)
**Violence type** *Physical* Pushing Beating Throwing objects Spitting Scratching Attack with sharp weapon (stick, knife,cutter, scissors) Attack with sticks/furniture Pinching Slapping Kicking Biting Suffocation *Emotional (psychological)* Threat of physical force Verbal abuse (name calling) Sexual harassment/threat	137 (24.8) 103 (18.6) 68 (12.3) 62 (11.2) 45 (8.1) 29 (5.2) 27 (4.9) 23 (4.2) 16 (2.9) 11 (2.0) 8 (1.4) 8 (1.4) 131 (23.7) 113 (20.4) 9 (1.6)
**Perpetrators** Patients’ relatives/friends Patients Colleague Hospital management	401 (72.5) 129 (23.3) 57 (10.3) 5 (0.9)

Table 4 shows that security personnel were alone in more than one-third of WPV events. In addition, more than two-fifths and more than one-third of violent events occurred during evening shifts and official vacation other than Friday, respectively. More than one-fifth of violent events were reported to the hospital administration. Only 3.8% of perpetrators received a verbal warning from the directors and only 2% of 553 reported events resulted in a lawsuit against the perpetrator.

**Table 4.** Circumstances, including time of event and to whom events are reported, of 553 workplace violence events reported by security personnel at a university hospital in Egypt.

	n (%)
**Security personnel working alone at time** **of event**	191 (34.5)
**Work shift at time of event** Morning shift (8am - 4pm) Evening shift (4 - 10pm) Night shift (10pm - 8am)	106 (19.2) 229 (41.4) 198 (35.8)
**Work day at time of event** Usual working days (Saturday to Thursday) Weekly vacation (Friday) Official vacations other than Friday	186 (33.6) 176 (31.5) 191 (34.5)
**Event reporting** To hospital administration To police To Nursing Syndicate	121 (21.9) 39 (7.1) 26 (4.7)
**Measures taken against perpetrators** Verbal warning from the directors Lawsuit against the perpetrator	21 (3.8) 11 (2.0)

Table 5 shows that participants reported that the commonest effect of violence was being bothered (80.9%), being fearful (69.5%), and having work dissatisfaction (61.8%) and anger/anxiety (47.3%). The commonest coping mechanisms were reporting to directors/supervisors (94.7%), telling family/friends (92.4%), pretending the event did not occur (77.1%) and replying to perpetrators themselves at the time of the event (74.0%).

**Table 5.** Consequences of workplace violence and coping mechanisms reported by reported by security personnel at a university hospital in Egypt in the past year (n=131).

	n (%)
**Consequence** Become bothered Become fearful Work dissatisfaction Have anger/anxiety Become irritable and watchful Become suspicious Decrease in performance and efficiency Absence from work/request for sick leave Lack of motivation Feel chronic fatigue/pain No effect Feel ashamed/guilty Plan to leave work/resign	106 (80.9) 91 (69.5) 81 (61.8) 62 (47.3) 53 (40.5) 49 (37.4) 33 (25.2) 32 (24.4) 28 (21.4) 19 (14.5) 19 (14.5) 13 (9.9) 6 (4.6)
**Coping mechanism** Reported to director/supervisor Told family/friends Pretended did not happen Replied the perpetrators Told a colleague Try to forget the event Transferred to other center/place of work Defend self physically No action	124 (94.7) 121 (92.4) 101 (77.1) 97 (74.0) 68 (51.9) 35 (26.7) 19 (14.5) 17 (13.0) 13 (9.9)

Categories are not mutually exclusive.

## Discussion

Hospital security workers, who are trained to offer tertiary prevention on escalation of an event, have also been acknowledged as a group at an increased risk of being assaulted ^12, 16^ and in need of tools to identify, alleviate, and avoid violent events in hospitals ^17^.

In this study, the majority (87.3%) of security personnel reported exposure to violence in the past year. A similarly high prevalence (63.8%) of type II violence, which is perpetrated by a client receiving services from an organization, such as a patient or guest, was reported among security guards and police officers in a study of six US hospitals ^16^. This high prevalence could be attributed to the nature of the work of security personnel, who repeatedly interfere in efforts to protect both staff and patient safety and are frequently injured in occurrences with violent patients ^18^. It has been previously acknowledged that hospital safety and security employees are often ignored in the healthcare violence-related injury literature ^9^. There are relatively few studies involving hospital security workers and these have revealed that these employees have some of the highest rates of violence-related incidences within hospitals, anywhere from 2 to 5 times as many incidences as nurses ^10– 12, 19– 21^. However, almost no studies have been carried out to discuss the risk and protective factors for hospital security employees ^9^.

In this study, being a woman and working for more than 5 years are independent predictors of violence exposure (AOR=7.7 and 10.1; respectively). Several studies reported that younger and less experienced security officers have elevated rates of violence-related incidences ^8, 12, 22– 25^.

In contrast to these findings, a study conducted in the Midwest US, showed that both age and gender were not associated with verbal violence, although, while healthcare workers more than 60 years old were less exposed to physical violence (odds ratio (OR) = 0.31, p<0.05) ^26^. In another study of US hospitals, the higher prevalence of WPV across all sub-types in workers of a younger age suggested that younger workers are more prone to be victims ^16^. Older workers may be more tolerant of these events resulting in their under reporting ^27^ or they may be more skilful at event de-escalation.

Most studies that investigated gender of recipients of violence-related incidences in healthcare reported that men are more liable to experience these injuries more than women ^11, 12, 23, 28– 30^, while others have not found difference in rates based on victims’ gender ^31, 32^.

The commonest suggestions to prevent violence in the current study were training (73.3%) and assigning a larger number of personnel (62.7%). The results of a survey of healthcare specialists from 19 hospitals in six cities of Heilongjiang Province, China are in agreement with our results, where the respondents exposed to WPV expected to obtain organizational and social support. Those exposed to psychological violence had a strong opinion of the need for targeted training to support their proficiency in responding to violence (OR = 1.319, 95% CI: 1.034–1.658) and endorsing WPV legislation (OR = 1.968, 95% CI: 1.523–2.543). Those exposed to physical violence incidences thought it might be helpful to strengthen staff with back-up support (OR = 3.101, 95% CI: 1.085–8.860) ^33^.

The commonest forms of physical violence in this study were pushing (24.8%), beating (18.6%) and throwing objects (12.3%). The threat of physical force and verbal abuse were the commonest emotional violence experienced (23.7% and 20.4%; respectively). Patients’ relatives/friends and patients themselves were the commonest perpetrators of violent events (72.5% and 23.3%; respectively). Similarly, verbal abuse (32.8%) was the most prevalent subtype of type II violence among security guards and police officers in the US followed by physical threat (24.1%), then finally physical assault (6.9%) ^16^.

Also, in the Emergency Department (ED) in Ankara, Turkey, regarding frequency of physical violence, security officers (75%) described higher rates than other professions working in the ED (p<0.0001); exposure to any form of verbal abuse was highest among housekeepers (90.9%) and security officers (90.6%). In that study, self-reported verbal threat and sexual harassment among security officers was 75% and 15.6%, respectively. Relatives or friends accompanying patients were most frequently responsible for the violence, and this confirms what was concluded in a previous study ^34^.

In a study within a large hospital system in Midwest US, nurses (OR = 1.87, p≤0.01) and security personnel (OR = 4.71, p≤0.01) reported verbal violent events in the past year more than any other job groups. Also, security staff (OR = 30.79, p≤0.001), registered nurses (OR = 2.72, p≤0.05), and mental health specialists (OR = 18.71, p≤0.01) were at a higher risk for physical violence ^26^. These findings concerning physical violence consolidate other previous studies that have documented security personnel, mental health specialists, and nurses as being at higher risk ^11, 16^.

In this study, security personnel were alone in more than one-third of events. Additionally, more than two-fifths and more than one-third of violent events occurred during evening shifts and official vacation other than Friday, respectively. More than one-fifth of violent events were reported to the hospital administration. Only 3.8% of perpetrator received a verbal warning from the directors and only 2% had a lawsuit against the perpetrator. In a study from Israel, security employees emphasized the contribution of the behaviour of physicians and nurses to the development of a violent incident, especially verbal interaction, and delayed response time. A previous study reported that clinical staff had a negative attitude towards security personnel and considered that calling them to situations is a contributing factor to the development of a violent episode, which can escalate the situation and should be avoided if not necessary ^2^.

In the present study, the commonest effects of violence were being bothered (80.9%), being fearful (69.5%), and having work dissatisfaction (61.8%) and anger/anxiety (47.3%). The commonest coping mechanisms were reporting to directors/supervisors (94.7%), telling family/friends (92.4%), pretending the event did not occur (77.1%) and replying to perpetrators (74.0%). Similarly, the most commonly reported reaction of ED staff in Turkey was sadness for sexual harassment (86%), physical assault (82.9%) and verbal threats (82%). The other reactions were anger for physical assault (83.8%), disappointment for verbal threat (79.3%) and disgust for physical assaults (69.3%). However, the most common coping method was “Doing nothing and keeping silent” (37.2–59.5%), followed by reporting to a manager (56.8%) for physical assaults, but this method is the least commonly used among participants experiencing verbal abuse (35.8%), verbal threat (34.7%) and sexual harassment (34.9%) ^34^.

### Limitations

As this is a small-scale study in a single hospital, its results cannot be generalized to all health facilities. The possibility of overestimating the problem by security personnel to gain sympathy or more incentives cannot be excluded.

From the results of this study, we recommend that security personnel at university hospitals need more training, and more adequate numbers should be recruited to cover shifts and time points with high risk of violence. The feasibility of other countermeasures suggested by security personnel need to be tested in further intervention studies before being adopted. Routine notification, analysis and record keeping of WPV events should be mandatory to monitor changes in the magnitude of the problem.

## Data availability

### Underlying data

Harvard Dataverse: Workplace violence against security personnel at a university hospital in Egypt, https://doi.org/10.7910/DVN/FRVSR2 ^35^.

This project contains the following underlying data:

- Datasheet containing all variables obtained from the questionnaire for all participants.

Data are available under the terms of the Creative Commons Zero "No rights reserved" data waiver (CC0 1.0 Public domain dedication).
