# Supplementary material for: Workplace violence against security personnel at a university hospital in Egypt: a cross-sectional study
Source: F1000Res. 2020 May 11;9:347. [Version 1] doi: 10.12688/f1000research.23252.1 (PMC7361496; doi:10.12688/f1000research.23252.1)
Supplement: Peer review history

# Peer review: Reviewer response for version 1

**DOI:** 10.5256/f1000research.25671.r65547

**Published:** 2020-07-14

**Authors:** Amal Hegazy

**Affiliations:** 1 Department of Community Medicine, Faculty of Medicine, King Abdulaziz University, Jeddah, Saudi Arabia; 2 Al-Azhar University, Cairo, Egypt

1. Security personnel are not considered one of the health care workers. The authors mentioned health care violence in the introduction, So, there is need to change.   
2. The authors mentioned the response rate among the study group in the result section, it is better to transfer it to method section.
3. In logistic regression model, Kindly could the authors answer this question, what are the variables that entered in the model? In other words, did the authors make an association between the exposure and the outcome first , then he takes the significant one in the model or what did they do?   

Is the work clearly and accurately presented and does it cite the current literature?

Yes

If applicable, is the statistical analysis and its interpretation appropriate?

Yes

Are all the source data underlying the results available to ensure full reproducibility?

Yes

Is the study design appropriate and is the work technically sound?

Yes

Are the conclusions drawn adequately supported by the results?

Yes

Are sufficient details of methods and analysis provided to allow replication by others?

Yes

Reviewer Expertise:

Occupational and environmental health topics

I confirm that I have read this submission and believe that I have an appropriate level of expertise to confirm that it is of an acceptable scientific standard.

# Peer review: Reviewer response for version 1

**DOI:** 10.5256/f1000research.25671.r63327

**Published:** 2020-05-29

**Authors:** Ibrahim Kabbash

**Affiliations:** 1 Public Health and Community Medicine, Faculty of Medicine, Tanta University, Tanta, Egypt

1. There is no need to mention the type of the study in the title.
2. The second paragraph in the introduction focused on health care violence. Security personnel can’t be considered as health care workers. The topic is violence in a health care setting rather than violence among health care workers. This issue should be considered in the whole part of introduction.
3. The authors did no mention the response rate among participants and whether there was any refusal to participate.
4. What is the justification of categorization of participant by age at 35 years?
5. In table 4, event reporting to nursing syndicate is not logic and strange. It is logic to be reported to authority that can deal with the accident. What is the authority of nursing syndicates to deal with accidents occurring at the entrance of the hospital or the wards?
6. In table 5 how the exposed worker to violence feel guilty? I can understand he/she may feel ashamed but being guilty is not clear to me.
7. In discussion the authors repeated again most of figures mentioned in the results section. They should discuss information and not repeated results in discussion

Is the work clearly and accurately presented and does it cite the current literature?

Yes

If applicable, is the statistical analysis and its interpretation appropriate?

Yes

Are all the source data underlying the results available to ensure full reproducibility?

Yes

Is the study design appropriate and is the work technically sound?

Yes

Are the conclusions drawn adequately supported by the results?

Yes

Are sufficient details of methods and analysis provided to allow replication by others?

Yes

Reviewer Expertise:

NA

I confirm that I have read this submission and believe that I have an appropriate level of expertise to confirm that it is of an acceptable scientific standard.
